# Pharma 4.0-Artificially Intelligent Digital Twins for Solidified Nanosuspensions

**DOI:** 10.3390/pharmaceutics14102113

**Published:** 2022-10-03

**Authors:** Christina Davidopoulou, Andreas Ouranidis

**Affiliations:** 1 Department of Chemical Engineering, Aristotle University of Thessaloniki, 54124 Thessaloniki, Greece; 2 Department of Pharmaceutical Technology, School of Pharmacy, Aristotle University of Thessaloniki, 54124 Thessaloniki, Greece

**Keywords:** digital twin, Pharma 4.0, nanosuspensions, spray drying, milling, micronization, solubility improvement, digital shadow, artificial neural networks, SAFT, Gibbs energy

## Abstract

Digital twins capacitate the industry 4.0 paradigm by predicting and optimizing the performance of physical assets of interest, mirroring a realistic in-silico representation of their functional behaviour. Although advanced digital twins set forth disrupting opportunities by delineating the in-service product and the related process dynamic performance, they have yet to be adopted by the pharma sector. The latter, currently struggles more than ever before to improve solubility of BCS II i.e., hard-to-dissolve active pharmaceutical ingredients by micronization and subsequent stabilization. Herein we construct and functionally validate the first artificially intelligent digital twin thread, capable of describing the course of manufacturing of such solidified nanosuspensions given a defined lifecycle starting point and predict and optimize the relevant process outcomes. To this end, we referenced experimental data as the sampling source, which we then augmented via pattern recognition utilizing neural network propagations. The zeta-dynamic potential metrics of the nanosuspensions were correlated to the interfacial Gibbs energy, while the density and heat capacity of the material system was calculated via the Saft-γ-Mie statistical fluid theory. The curated data was then fused to physical and empirical laws to choose the appropriate theory and numeric description, respectively, before being polished by tuning the critical parameters to achieve the best fit with reality.

## 1. Introduction

A digital twin is the mirrored functional space model of a manufacturing unit that responds to physical state changes which is purposed for surveillance, optimization and prediction [1].The depth of the replication appointment to the physical asset by the digital model space remains ambiguous, hence various integration levels occur, whilst optimality appears controversially dependent of the corresponding functionality, complexity and availability of infrastructures [2]. The birth of the digital twin pertained to a crafty inspiration of the NASA Apollo 13 mission controllers 52 years ago. In a need-based, lifesaving attempt, the crew set up and modified simulations to mimic the multiplicity of the spacecraft’s physical conditions, those which occurred 45,000 miles from the Earth’s surface. This first successful approach spurred inspiration across applied scientific fields.

Since then, digital modeling is emancipating the state-of-the-art approach for delivering process and product lifecycle awareness, enabling unparalleled plantwide control, optimization, and prediction for material manufacturing [3]. Akin to such product material ontologies, artificial neural networks (ANNs) have ascended as a surrogate, responsive framework, poised to identify and simulate non-linear dependencies between digital twin variables [4]. As such, ANNs constitute the requirements for demanding, multiple, complex experiments towards the generation of the product manufacturing cycle (PML) data, irrelevant. Moreover, ANNs recognize the relationship patterns between independent and dependent variables of the PML, whether the available data set is insufficient or noisy [5]. The latter advancement proves to be of extreme importance, hence such variables lie at the core of the digital twin mechanistic and/or empirical algorithms, whilst poor data set availability effects common burdens when dealing with expensive raw and starting materials, such as those handled by the pharma industry. Successful applications of the ANNs in the field include the calculation of API’s critical quality attributes and their physicochemical properties, the prediction of the in-vitro drug release profile, the identification of raw material–tablet properties characterization, the dissolution behavior, and the size effect of injectable microparticle prediction [6].

For such a heavily regulated sector, ANN-fused artificially intelligent digital twins might accurately predict the critical process parameters, the final product’s quality attributes, integrate the process steps and lead seamless scale-up studies. The advantages of possessing this blend of stochastic, empirical, and mechanistic knowledge becomes invaluable in terms of technical, economical and risk eliminating factors by the creation of novel, in-silico intelligent analogues to the physical product. Counterintuitively, although digital twin applications could transform the pharmaceutical industry, whereas precision and risk elimination are evidently required, the penetration of these Pharma 4.0 tools appears limited. In addition, artificially intelligent, advanced digital twin systems, i.e., ANNs-fused digital twins, have not been reported by the field’s literature.

Under this lens, arguably, one significant contemporary technological challenge of the pharmaceutical industry is to improve solubility and in vivo dissolution profiles of poorly soluble active pharmaceutical ingredients (APIs). To address this challenge, APIs are mixed with stabilizers and formulation excipients composites and comminuted by wet media milling into nanosuspensions. The obtained, coated, liquid crystals are further stabilized by spray drying solidification to allow for further processability. Taking into account the proven reliability and scalability of this method, we introduced novel thermodynamic models to calculate such composite material solubility by assessing the obtained stabilizer-coated nanoparticle Gibbs energy anisotropic minimization, quantified by the implementation of PC-SAFT interrogations coupled with elastic tensor analysis [7].

**Figure 1:**
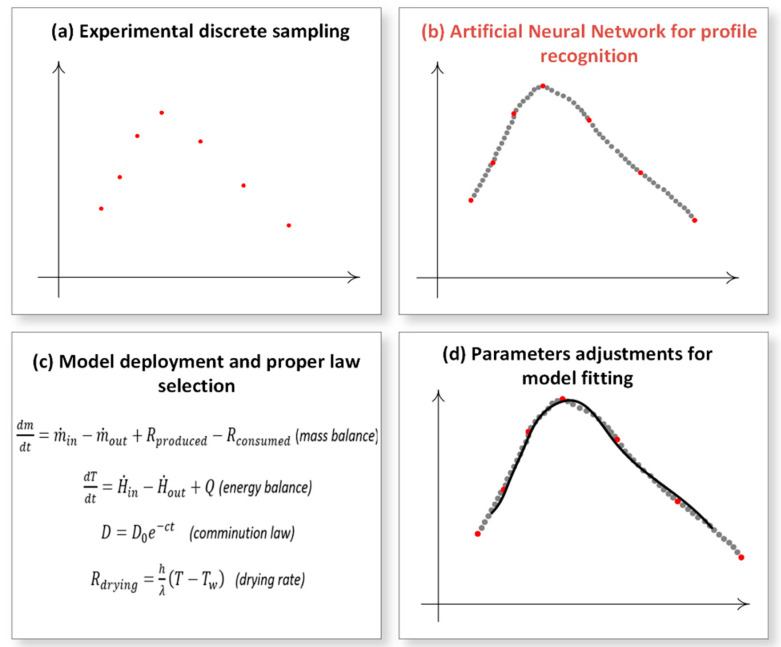
Digital twin deployment and parameter estimation strategy by steps: (**a**) real data sampling from the physical object, (**b**) data multiplication via pattern recognition using artificial intelligence algorithms aiming accuracy enhancement, (**c**) determination of the proper descriptive physical and empirical laws, and (**d**) adjustment of tuning parameters to achieve best curve fit.

In this current research, we build on these foundations to create a novel, fully operational, neural network-intelligent digital twin, capable of describing the course of manufacturing of solidified nanosuspensions given a defined PML launching chronic point, and also predict and optimize the engaged process outcomes [2]. Elaborating on this strategy, the ANN was embedded in a stepwise manner among data sampling, model deployment and curve fitting towards the implementation of the digital twin (Figure 1). The ANN fulfilled the mission of augmenting the abundance of the available discrete data generated to calibrate and validate the model whilst eliminating the experimental burden and the model’s uncertainty [8].

## 2. Materials and Methods

### 2.1. Process Design

The API’s formulation processes, including wet milling and spray drying steps, were modelled using the Siemens gProms Formulated Products^®^ platform Process Systems Enterprise, gPROMS, (www.psenterprise.com/products/gproms, accessed on 2 August 2022). Before formulation, it is crucial to select the compatible polymer or surfactant to coat the liquid crystals, the one which best enhances the API’s dissolution performance [9]. This pivotal step was included in the process model; the selection was based on the implementation of the computational statistical associating fluid theory criteria (PC-SAFT) to substitute for the experimental trials.

In detail, the stabilizer’s addition generates a semi-solid interface that adds to the dissolution’s Gibbs energy decrease by *GEE* (J mol^−1^), according to Equations (1)–(3) [7].

(1) $$GEE=G_{m}^{s}+G_{m}^{i}=RTln\frac{K_{2}}{K_{1}}$$

(2) $$G_{m}^{s}=\frac{2\gamma V_{m}}{r}\left(1-\frac{C}{r}\right)$$

(3) $$G_{m}^{i}=1.7\frac{\varepsilon_{API}\sigma_{API}}{\rho_{stab}\Delta \left(\sigma_{stab-API}\right)\left(\varepsilon_{stab-API}\right)m_{stab}}\frac{\gamma V_{m}}{r}$$

The $G_{m}^{s}$ and $G_{m}^{i}$ terms refer to the particle’s surface and the interface caused by the stabilizer Gibbs energy. Where $\frac{K_{2}}{K_{1}}$ is the ratio of the dissolution equilibrium coefficient post and prior the powder’s size decrease respectively, *T* (K) is the dissolution temperature and *R* (J mol^−1^ K^−1^) is the universal gas constant. In Equation (2) *γ* (Ν m^−1^) is the surface tension, *V_m_* (m^3^ mol^−1^) the API’s molar volume, *r* (m) the particle’s characteristic size and *C* (m) a parameter equal to $1.5{\left(\frac{V_{m}}{N_{A}}\right)}^{\frac{1}{3}}$, while in Equation (3) Δ (m) is the material’s distance between the molecular layers, *m_stab_* (−) the stabilizer’s number of segments per chain based on PC-SAFT theory, and *ε* (eV) and *σ* (m) are the depth of pair potential and the segment diameter, respectively. The terms *ε_stab-API_* and *σ_stab-API_* were calculated using the Berthelot-Lorentz combining rules. Stabilizer candidates were chosen, namely Poloxamer-188, Poloxamer-407 and HPC-SL. As far as poloxamers are concerned, since both are copolymers containing ethylene oxide (EO) and propylene oxide (PO) groups in different proportions, the component’s PC-SAFT parameters were calculated using Berthelot-Lorentz rules (see Table 1).

The polymer that contributes dominantly to the Gibbs energy decrease is the one to be chosen as the stabilizer. Gibbs energy is not calculated experimentally, yet on the other hand the zeta-dynamic potential can be correlated to the interfacial Gibbs energy. The physical parameter estimations regarding the API and the stabilizers were performed using the gProperties^®^ package of Siemens Process Systems Enterprise (https://www.psenterprise.com/products/gproms/properties, accessed on 2 August 2022). The Saft-γ-Mie equations of state were utilized to calculate the temperature dependence on the density and the heat capacity of the components.

The wet milling process model was based on the main batch grinding Equation (4) and three supplementary empirical grinding functions, each of them complemented by their corresponding breakage rate. This batch grinding mass balance set up explains the change of the powder’s mass fraction containing particles of size interval *i*:

(4) $$\frac{dw_{i}}{dt}=\sum_{j=1}^{i-1}\left[S_{j}w_{j}\frac{d\left(b\left(i,j\right)\right)}{dx_{i}}\right]-S_{i}w_{i}\;$$

The initially examined breakage function was the one proposed by de Vegt et al. [15], and it is referred to as the de Vegt model (Equation (5)). The corresponding breakage rate is strongly dependent on the product’s material properties (Equation (6)):

(5) $$b\left(i,j\right)=\frac{S_{i}}{S_{j}}$$

(6) $$S\left(i\right)=c\;\frac{E_{kin,i}E_{fract,i}\sqrt{\frac{P_{y}}{\rho }}}{VH\sqrt{x_{i}}K_{1c}}$$

(7) $$E_{kin,i}=W_{m,kin}\rho V_{i}$$

(8) $$E_{fract,i}=0.896{\left(\frac{\pi \left(1-\upsilon^{2}\right)}{\Upsilon }\right)}^{\frac{2}{3}}{\left(0.0183\delta^{2}{\left(\frac{V_{0}}{V_{i}}\;\right)}^{1/4}\right)}^{5/3}$$

In Equations (5)–(7), *S(i)* (s^−1^) is the breakage rate of a particle of size interval *i*, *E_kin_* (J) and *E_fract_* (J m^−3^) are the kinetic energy of the particles and the fracture energy, respectively, *P_y_* (Pa) is the yield pressure, *ρ* (kg m^−3^) is the particle’s density, *V* (m^3^) is the mill’s chamber volume, *H* (Pa) is the particle’s hardness, *x_i_* (m) is the particle size *i*, *K_1C_* (Pa m^−1/2^) is the stress intensity factor, *W_m,kin_* the mass specific impact energy (J kg^−1^) and *b(i,j)* (−) is the mass fraction of the product that fell from size interval *j* to *i*. In Equation (8), *δ* is the solubility parameter (Pa^1/2^), *V_i_* (m^3^) is the particle’s volume, *υ* (−) is the Poisson’s ratio, *Υ* (Pa) is the Young’s modulus of elasticity and *V_0_* (m^3^) is the unit’s crystal volume. While all the other parameters in Equations (6) to (8) are pre-estimated, the breakage rate parameter *c* (−) is a tuning parameter, estimated by experimental data. The second breakage function and breakage rate used for the wet milling simulations is proposed by Austin et al. [16] and it is referred to as the Austin model (see Equations (9) and (10)):

(9) $$b\left(i,j\right)=\varphi {\left(\frac{x_{i}}{x_{j}}\right)}^{\gamma }+\left(1-\varphi \right){\left(\frac{x_{i}}{x_{j}}\right)}^{\beta }$$

(10) $$S\left(i\right)=\{\begin{array}{c} \;\;\;\;a{\left(\frac{x_{i}}{x_{crit}}\right)}^{d}\;\;\;x_{j}\geq x_{crit}\\ \;\;\;\;\;\;\;\;\;\;0\;\;\;\;\;\;\;\;x_{j}<x_{crit}\; \end{array}$$

where *φ*, *γ*, *β*, *d* (−) and *a* (s^−1^) are the tuning parameters, while *x_i_* and *x_j_* are the product’s and the post-breakage final particle size accordingly (m), and *x_crit_* is the critical particle size, namely the size after which no breakage occurs. The last breakage function was the one proposed by Kapur et al. [17] and it is referred to as the Kapur model (model Equations (11) and (12)):

(11) $$b_{i,j}={\left(\frac{x_{i}}{x_{j}}\right)}^{e}$$

(12) $$S_{i}=Ax_{i}^{k}$$

where *e, k* (i) and *A* (s^−1^) are the tuning parameters. Apparently, all three breakage functions and rates encumber tuning and physical parameters respectively, with Austin presenting the highest number of considered tuning parameters and de Vegt the lowest. The parameter estimation was conducted in the Siemens Process Systems Enterprise gPromsFP^®^ Model Validation platform (https://www.psenterprise.com/products/gproms/modelbuilder, accessed on 2 August 2022), applying the Maximum Likelihood Estimation method. In the spray drying process, the droplets’ hydrodynamic diameter was assumed to obey lognormal distribution and for the drying rate calculation the Oakley’s model was adapted [18]. For each particle size interval *i* was defined from the particle size distribution and was dispersed in a droplet, which in turn belongs to a size interval *z*, the local mass and energy balance describing the spray drying model which is described respectively by the Equations (13) and (14).

(13) $$-{\dot{m}}_{s,i,z}\frac{dx_{i,z,j,t}}{dt}={\dot{N}}_{i,z,j,t}$$

(14) $${\dot{m}}_{s,i,z}\left(C_{p,s,i}+\sum_{j=1}^{N_{j}}x_{i,z,j,t}C_{p,j}\right)\frac{dT_{i,z}}{dt}=hA_{i,z}\left(T_{g}-T_{i,z}\right)+{\dot{m}}_{s,i,z}\sum_{j=1}^{N_{j}}\lambda_{j}\frac{dx_{i,z,j,t}}{dt}\;$$

where ${\dot{m}}_{s,i,z}$ (kg s^−1^) is the corresponding solids particles flowrate, $x_{i,z,j,t}$ (kg kg^−1^) is the dry basis moisture content of the liquid specie *j*, ${\dot{N}}_{i,z,j,t}$ (kg s^−2^) is the drying rate time derivative, $C_{p,s,i}$ (J kg^−1^ K^−1^) is the solid material’s specific heat capacity and $C_{p,j}$ the liquid specie’s corresponding one, $T_{i,z}$ (K) is the solid particle’s temperature, *h* (J m^−2^ s^−1^ K^−1^) is the heat transfer coefficient, $A_{i,z}$ (m^2^ s^−1^) is the shrinking rate of the surface area of the droplet, $T_{i,z}$ (K) is the droplet’s temperature, and *λ_j_* (J kg^−1^) is the latent heat of vaporization of the liquid specie *j*. The local vapor phase’s mass balance for the evaporated liquid specie *j* and the vapor’s phase energy balance is described in Equations (15) and (16), respectively.

(15) $$\frac{dm_{v,j}}{dt}={\dot{m}}_{v,in}x_{v,j,in}-{\dot{m}}_{v,out}x_{v,j,out}+\sum_{i=1}^{N_{i}}\sum_{z=1}^{N_{z}}\int_{t=0}^{t_{\tau }}{\dot{N}}_{i,z,j,t}dt$$

(16) $$\frac{dH_{v}}{dt}=H_{v,in}-H_{v,out}+\sum_{i=1}^{N_{i}}\sum_{z=1}^{N_{z}}\sum_{j=1}^{N_{j}}\int_{t=0}^{t_{\tau }}{\dot{N}}_{i,z,j,t}dt\;C_{p,v,j}\left(T_{v}-T_{0}\right)-\sum_{i=1}^{N_{i}}\sum_{z=1}^{N_{z}}h\int_{t=0}^{t_{\tau }}A_{i,z,t}\left(T_{v}-T_{i,z}\right)dt$$

where ${\dot{m}}_{v,in}$ and ${\dot{m}}_{v,out}$ (kg s^−1^) are the inlet and outlet mass flowrate of the vapor phase respectively, $x_{v,j,in}$ and $x_{v,j,out}$ (kg kg^−1^) are the inlet and outlet mass fractions of the liquid specie j in the vapor phase respectively, $H_{v,in}$ and $H_{v,out}$ (J s^−1^) are the inlet and outlet enthalpy flowrates of the vapor phase respectively and $C_{p,v,j}$ (J kg^−1^ K^−1^) is the specific heat capacity of liquid specie j in the vapor phase, *t_τ_* (s) is the droplets residence time inside the spray dryer’s chamber and *T_v_* (K) is the vapor phase’s temperature. The unhindered drying rate *Ν_u,z,j_* (kg s^−1^), i.e., the drying rate of the very same droplets without containing solids described by Equation (17) and is interlinked to the actual drying rate by the relative drying rate *f* (−) (Equation (18)).

(17) $${\dot{N}}_{u,z,j,t}=\frac{h}{\lambda_{j}}A_{i,z,t}\left(T_{v}-T_{wb,j}\right)$$

(18) $${\dot{N}}_{i,z,j,t}=f{\dot{N}}_{u,z,j,t}$$

where *T_wb,j_* (K) is the wet bulb temperature of the liquid specie *j*.

### 2.2. Experimental Study and Digital Twin Thread Structuring

The wet milling process of the API Itraconazole nanosuspension was performed by a Pulverisette 7 Premium (Fritsch GmbH, Idar-Oberstein, Germany). Delivered discrete experimental values [19] were first used to train an ANN and form a complete size reduction profile (see Table 2), while afterwards this profile is used as a basis to fit the tuning parameters of the digital twin model. The process reduced the powder’s particles’ sizes approximately by one order [8]. Post wet milling, the micronized API suspension was spray dried to remove the liquid phase and obtain the desired dry powder product [19]. The spray dryer used in the experiment was the Büchi B-191 Mini Spray dryer (Büchi, Flawil, Switzerland).

The technical specifications of the mill’s mechanical parameters, such as the rotation and the revolution speed, the capacity and the equipment’s volume, were determined by the manufacturer. Moreover, the specific ranges of input data were adopted by the manufacturer’s technical specification sheet of the Buchi B191 Mini Spray dryer (Büchi, Switzerland). Both data input sets are herein demonstrated in Table 3.

The system descriptive equations were concluded by Section (B), containing linear and differential equations. For instance, the Equations (13)–(18) were considered as a system of six non-linear algebraic equations:

(19) $$f\left(t,\;{\dot{x}}_{i,z,j,t},\;{\dot{N}}_{i,z,j,t}\right)=0\;\mathrm{Equation (13)}$$

(20) $$f\left(t,x_{i,z,j,t},{\dot{x}}_{i,z,j,t},T_{i,z},{\dot{T}}_{i,z}\right)=0\;\mathrm{Equation (14)}$$

(21) $$f\left(t,{\dot{m}}_{v,j},N_{i,z,j,t}\right)=0\;\mathrm{Equation (15)}$$

(22) $$f\left(t,{\dot{H}}_{v},N_{i,z,j,t},T_{i,z}\right)=0\;\mathrm{Equation (16)}$$

(23) $$f\left(t,{\dot{N}}_{u,z,j}\right)=0\;\mathrm{Equation (17)}$$

(24) $$f\left(t,{\dot{N}}_{u,z,j},\;{\dot{N}}_{i,z,j,t}\right)=0\;\mathrm{Equation (18)}$$

The system’s variables embedded first order derivatives and were calculated utilizing finite difference approximations. Specifically, the Backward Differentiation Approximations (BDF) were adopted, which present a universally approved method to approach DAE system solutions [20]. By applying the BDF using a time step size *h_s_*, at time *t_n_*, the DAE system transforms into a linear system featuring six equations bearing six unknown variables, each derivative of them being approximated as in Equation (25).

(25) $$\;{\dot{x}}_{i,z,j,t_{n}}=h_{s}^{-1}\sum_{l=0}^{k}a_{l}x_{i,z,j,t_{n-l}}$$

where *k* is the degree of the interpolating polynomial and *a_l_* is the *l*th polynomial’s level coefficient.

### 2.3. Integration of Artificial Neural Networks for Parameter Tuning

The ANN was properly trained to simulate the dynamic comminution profile inside the physical mill. Data sampling during the wet mill process is a difficult task, hence it requires temporary terminations. In addition, the final crystal size itself is not adequate for the characterization of the mill’s performance. It is therefore crucial to obtain additional information of the dynamic profile in order to fulfil the purposed optimization and prediction purposes. The discrete experimental training data includes the D50 values taken after sampling in between 6-min intervals. The transfer function used for the hidden layers was the Sigmoid Function (Equation (26)), due to its ability to identify non-linear relationships. For the output layer, the transfer function selected was the Rectified Linear Unit (ReLU), hence the case was addressed through a regression scenario and not as a classification (Equation (27)). The ANN training adopted the error backpropagation methodology towards defining the weights’ adjustments, but its layout was forward propagating. Furthermore, the weights *w_i,j_* connecting a layer with *n_j_* neurons with the next layer with *n_i_* neurons, followed the He initialization (Equation (28)).

(26) $$f\left(x\right)=\frac{1}{1-e^{-x}}$$

(27) g(x)=max (0,x)

(28) $$w_{i,j}\propto \;\sqrt{\frac{2}{n_{j}}}\;\;\;\;\;i\in \left(1,n_{i}\right)\;,\;\;j\in \left(1,n_{j}\right)$$

For the identification of the D50(t) profile, polynomial regression was used with high efficiency, especially whereas polynomial fitting is of a higher order (≥4).

## 3. Results

### 3.1. Material Critical Quality Attributes and the Material System’s Interfacial Gibbs Energy Assesment

**Figure 2:**
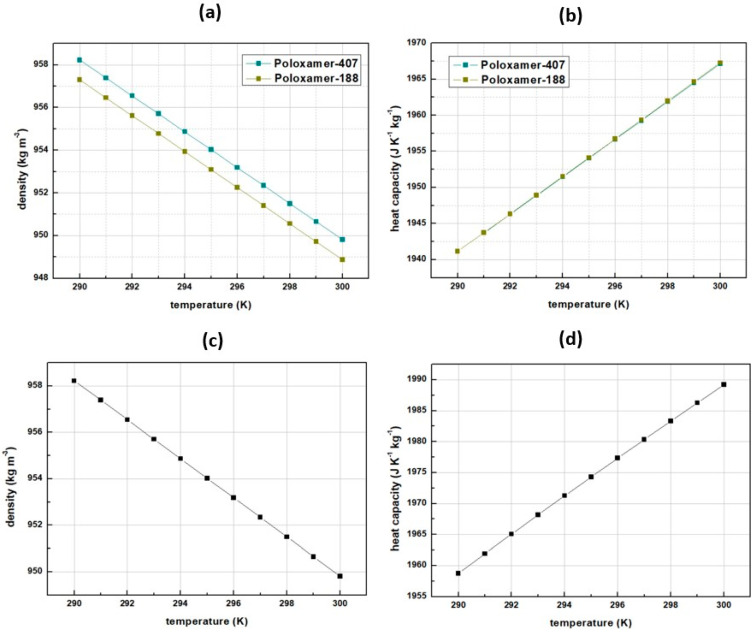
Saft-γ-Mie stabilizer candidates, material properties results (**a**) Poloxamers’ densities (**b**) Poloxamers’ heat capacities (**c**) HPC-SL density (**d**) HPC-SL heat capacity as a function of temperature.

The density and the heat capacity temperature profile of the pure stabilizers, calculated via the Saft-γ-Mie statistical fluid theory, are shown in Figure 2. Poloxamer-188 material poses the lowest density and the lowest heat capacity, while HPC is denser than poloxamers. Calculations of the pure stabilizers’ densities were pivotal for the determination of the stabilizer’s interfacial Gibbs energy. Apparently, density effects the intermolecular forces of the stabilizer-API composite (see Table 4). For higher temperatures, the composite’s cohesive forces decrease as the kinetic energy of the molecules increases, and thus they tend to escape their structured positions [21]. For the same reason, when temperature increases the material tends to expand, the density decreases, and the corresponding particle’s surface tension *γ* (Ν m^−1^) decreases as well, see Equation (29) [9]. In addition, the zeta-potential bourn within the semi-solid interface that the stabilizer’s addition forms [22], is utilized as indicator of its efficiency (Table 3). A stabilizer causing high absolute values of zeta-potential, creates strong repulsive forces (Coulomb forces) preventing the particles from aggregating.

(29) $$\gamma =-0.33k_{B}T{\left(\frac{N_{A}\rho }{Mr}\right)}^{\frac{2}{3}}\left[\ln\left(\frac{S_{0}}{55.6}\right)+5\right]$$

In Equation (29), where *k_B_* (J K^−1^) is the Boltzmann constant, *Mr* (g mol^−1^) and *ρ* (g m^−3^) are respectively the molecular weight and the density of the particle, *N_A_* (mol^−1^) the Avogadro number and *S_0_* (−) the solubility of the pure API in water in absence of the stabilizer.

According to the results shown in Table 4, the free Gibbs energy of the interface is correlated both to the density and the zeta-potential. The decrease of surface density and tension enhances the binding with the stabilizer, which in turn plays a crucial role in the zeta-potential absolute value increase [23]. While Gibbs energy is a measurement of the maximum non-expansion work available in a system, interfaces of higher Gibbs free energy favors the effects of the electrostatic Coulomb forces. Among the three investigated stabilizers, Poloxamer-188 presents the highest interfacial Gibbs energy, and as a result the highest contribution in the dissolution Gibbs energy enhancement (Equation (1)) and the highest absolute zeta-potential, making it suitable for selection.

### 3.2. Parameter Fitting

**Figure 3:**
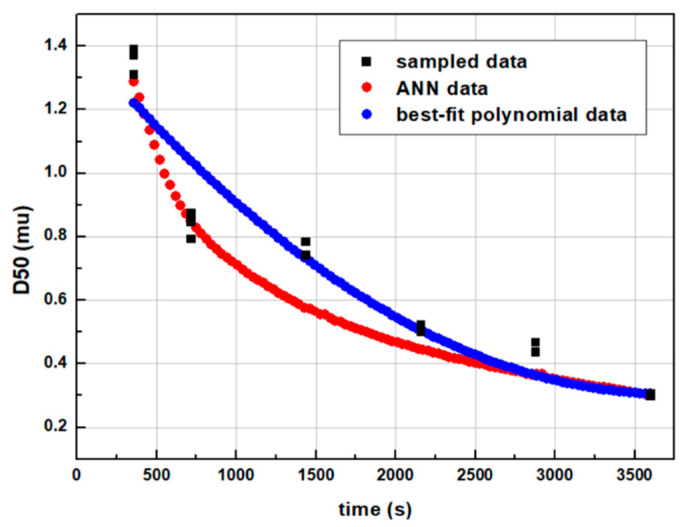
Wet milling dynamic comminution profile of experimental data points, ANN generated and best-fit polynomial curve.

The results of the milling’s dynamic profile mapping appear in Figure 3. The artificial neural network achieved fitting within 1.6% mean squared error (MSE), while the second-order polynomial achieved fitting with 1.1% MSE. Although using polynomial fitting provides a bit lower percentage of MSE, the advantages of using ANNs for pattern recognition are numerous, as discussed in the introduction section. Also, the discrete experimental data points appear to be noisy. Deep neural networks identify noisy data rather than memorize it and include it in the training process [24]. However, there exist data fusion algorithms with insignificant computational requirements used to minimize noise during sampling, such as the Extended Kalman filter.

**Figure 4:**
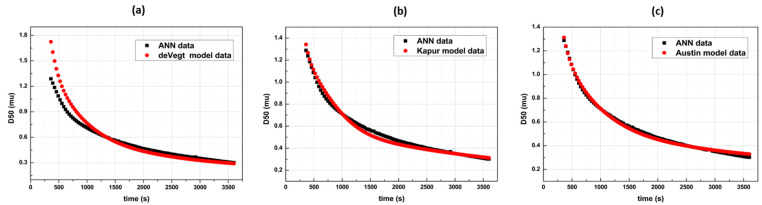
Result dynamic profiles for the three breakage functions (**a**) de Vegt function’s best fit curve generating a 0.89% MSE (**b**) Kapur function’s best fit curve generating a 0.08% MSE, and (**c**) the Austin function’s best fit curve generating a 0.02% MSE.

The Maximum Likelihood Estimation best-fit results comparing the three breakage functions are shown in Figure 4. The Austin model provided the best experimental fit with the lowest MSE, while the de Vegt model provided the highest one. Furthermore, as discussed above, the Austin model’s breakage function included more tuning parameters than the rest, with the Kapur model being the second and the de Vegt model being the last one. Considering this fact, it was found that the polyparametricaly tuned model exhibited a suitable experimental fit curve. As expected, the integration of numerous tuning parameters enhances the curve fitting performance, especially when it desired to interpolate complex experimental data profiles. The tuning parameter herein successfully plays the role of “correction factor”.

### 3.3. Sensitivity Analysis

#### 3.3.1. Wet Milling

**Figure 5:**
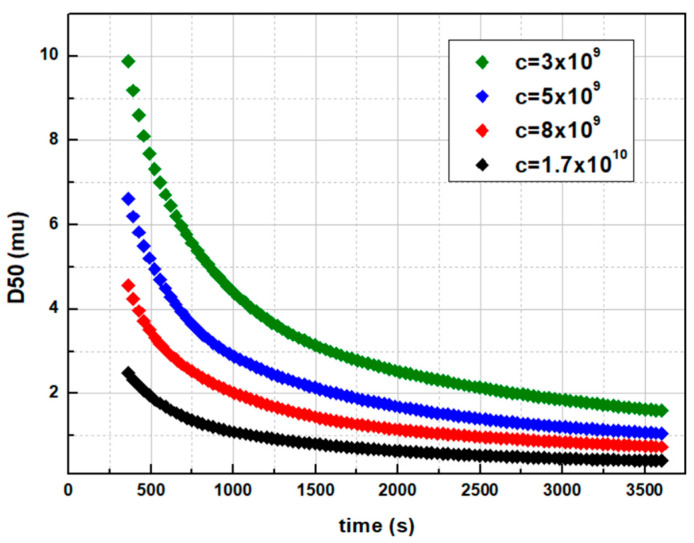
Breakage parameter c effect on the size comminution profile.

A sensitivity analysis was performed against the various considered tuning parameters regarding the breakage functions conducted to examine their effects on the model outputs. Figure 5 shows the effect of the de Vegt model’s breakage rate parameter *c* (−) on the final D50 size profile. Parameter c is the tuning parameter existing in the model, while the others are predetermined according to the material’s and the mill’s characteristics (see Equations (6)–(8)).

The de Vegt model’s breakage distribution function remained the same during the analysis as when combining Equations (5) and (6) the final form appears dependent on the $\frac{xi}{xj}$ size reduction ratio (Equation (30)).

(30) $$b\left(i,j\right)={\left(\frac{x_{i}}{x_{j}}\right)}^{1.25}$$

**Figure 6:**
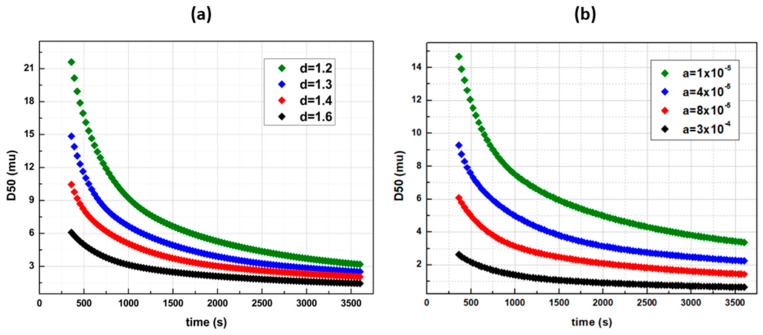
D50 time profiles with Austin’s breakage function in various *a* and *d* parameter values with *γ* = 1.17 and *φ* = 0.3 (**a**) *a* = 8 × 10^−5^ and (**b**) *d* = 1.6.

**Figure 7:**
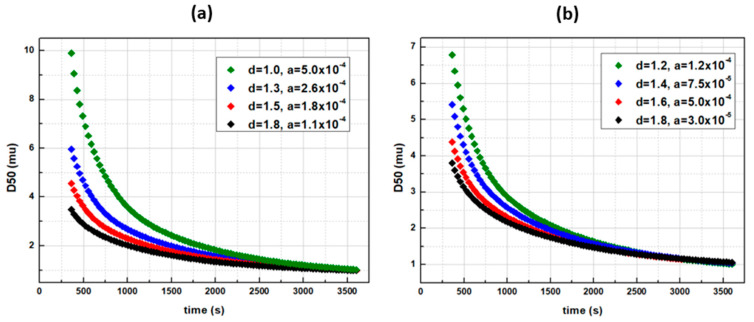
Austin’s breakage function for a D50 = 1 μm final size via various time profile paths with (**a**) *γ* = 1.17 and *φ* = 0.3 and (**b**) with *γ* = 1.25 and *φ* = 1 (de Vegt approximation).

Apparently, increasing *c* provides higher breakage rates for larger particles and results in efficient comminutions. However, with the latter featuring the only tuning parameter for a given API, each desired final size shall unveil its own unique reduction profile. De Vegt model’s breakage function is ideal for qualitative analysis cases, e.g., when only the initial and the final sizes are of the main interest. In Figure 6 and Figure 7, Austin’s comminution profiles are illustrated, based on the breakage rate’s sensitivity analysis. The effects of the tuning parameters *a* (s^−1^) and *d* (−) were interrogated, and it was found that by increasing each or both of *a* and *d*, the breakage rates of interval sizes *i* (Equation (10)), and thus the comminution efficiency, increases (Figure 6). The breakage distribution function *b(i,j)* during the Austin model’s relative sensitivities remained the same, with *φ =* 0.3, *γ* = 1.17 and *β* = 4. In comparison to de Vegt model, a desired D50 size is achieved via various profile paths (Figure 7a). In addition, even when *φ* = 1 and *γ* = 1.25, namely when the distribution function *b(i,j)* is the same with the de Vegt’s one, the existence of the exponent *d* as a secondary tuning parameter in the *S(i)* calculation function allows the formation of multiple profiles leading to the same result (Figure 7b). This is the benefit of engaging multiple tuning parameters within the breakage rate function, as they render the digital twin capable of fitting multiple profiles.

**Figure 8:**
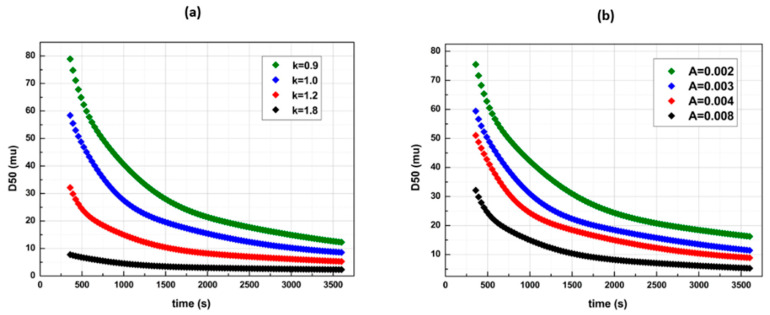
D50 time profiles with Kapur’s breakage function in various *k* and *A* parameter values with *e* = 6 (**a**) *A* = 0.008 and (**b**) *k* = 1.2.

**Figure 9:**
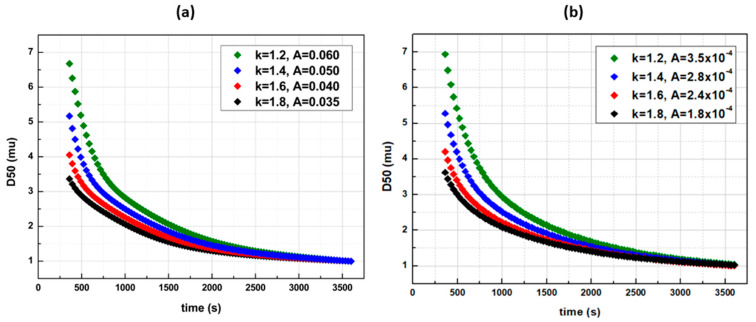
Kapur’s breakage function for a D50 = 1μm final size via (**a**) various time profile paths with *e* = 6 and (**b**) various profile paths with *e* = 1.25 (de Vegt approximation).

Figure 8 and Figure 9 illustrate the Kapur model D50 time profile results (*e* = 6), following the same strategies. The Kapur model proved to be a sufficient breakage function as it recognizes multiple time profiles. Nevertheless, the *b(i,j)* distribution function contains one tuning parameter in comparison with the Austin model, and this is the reason that effective experimental fit accuracy cannot be approached by the theory.

#### 3.3.2. Spray Drying

**Figure 10:**
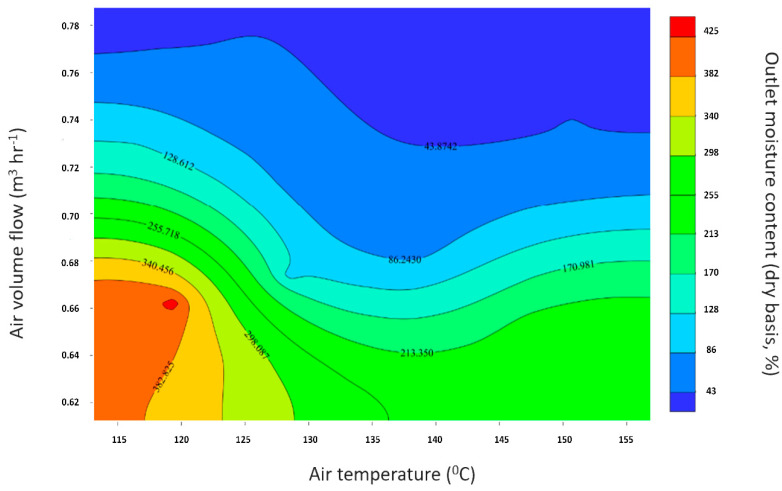
Contour sensitivity analysis diagram of the spray drying model.

Two-factor sensitivity analysis was conducted to the spray drying model to map the design space. Figure 10 presents the obtained analysis’s contour diagram, whereas the factors considered are the air temperature and the air volume flow rate, while the response is the final product’s humidity, which constitutes the critical quality attribute related to the process. The contours below describe the final moisture content of the product decrease as the air’s temperature and/or its volumetric flow rate increases for the given initial dry humidity (g g^−1^). The air water capacity threshold rises proportionally with temperature when the temperature increases the air’s given humidity (g g^−1^) covering a lower proportion of the moisture threshold and its relative humidity decreases, allowing humidity absorption [7].

## 4. Discussion

In our artificially intelligent digital twin thread, comminution efficiency appears dependent both on the material system selection and on the equipment’s parameter settings. The relevant capacity and the physical properties of the API and of the grinding media were included in the digital twin’s working parameters, defining the quality attributes of the final product. Therefore, this digital twin thread is capacitated to analyze what-if equipment and material scenarios for early production assessments towards the reduction of material waste and the optimization of time schedule and process functions serving multiple batch mill units [25]. In addition, the algorithm is poised to identify irreversible faults and incompatibilities beforehand, for the related processes and the material system (e.g., over-efficient comminution profiles, API’s unwanted loss by dissolution during milling etc.) and propose controller actions to avoid them. Spray drying is a complex procedure, crucial for the final API’s powder formation. It is a continuous process enabling real-time data sampling and immediate response to changes of the physical object’s properties. In our digital twin, the spray drying process is described as a multi-parametric block, since the final temperature and moisture content of the product depend on the drying air’s temperature, pressure, and capacity as far as the initial product’s flow, temperature, and droplet size distribution. Therefore, it can predict the drying efficiency given the air temperature specification, hence apart from the convergent mass and energy balances it exploits psychrometric calculations to determine the air’s relative humidity and its moisture capacity threshold [7], thus being advantageous in delivering realistic analysis reports.

Our algorithm may receive real-time data and return forecasts of the temperature profile inside the spray dryer for any given initial input conditions, an extremely important operation, hence the product is required to be obtained in a stable crystalline state, while its temperature should not overcome the API’s and the excipients’ melting point [26,27]. This digital twin ameliorates any scale-up related efforts and product degradation risks, while contributing to the optimization of the process outlining the system’s design spaces. In this recursive digital model, the proper empirical law selection, the one that more accurately describes the physical object’s progress, is dependent on the crowd of its tuning parameters, namely the parameters acceptable to be adjusted within boundless values towards improvement of the model’s prediction accuracy. Considering processes like wet milling, although the material and energy balances invoke fundamental laws of physics, models that describe specific functions, such as the comminution laws, must include data-driven parameters. Under this lens, for this specific unit block, using trained neural networks could replace such models efficiently, although limitations should be considered, and those lie in the idiosyncratic nature of the methods such as the ability of the empirical models to point out relative dependencies between variables [28]. ANNs identify patterns without obeying a strict mathematical function, thus generalizing the relationship between independent-dependent variables. Both ANNs and polynomial fitting are efficient, depending on the circumstances chosen. In addition, as the adjective “empirical” implies, experiments cannot be avoided completely, hence even the structure of the mathematical models requires real data for their parameter training.

Contemplating the limitations of the integration depth of our approach, there exist cases where a specific level of uncertainty is required, for instance when model discrepancy needs to become recognized. A model can simultaneously be mechanistically biased, including over-confident parameter estimates, and therefore can also be effected by model discrepancy. In this research, the adjusted parameters were not predetermined, hence they do not possess physical status, yet their existence lies within calibration purposes, such as fitting experimental data towards the enhancement of the digital twin’s validity. Such tuning parameters, although as iterated irrelevant of physical interrelations, become scientifically vital, whereas complex process simulations are considered, as they aid the translation of the data set ontologies to the related physical property [8]. Adjusting tuning parameters to discrete data points vertically amplifies the model’s data value and therefore improves overall accuracy as uncertainty, and its minimization, shall be in frame with the output results.

The digital twin thread requires input modifications when APIs other than Itraconazole are considered. Specifically, different APIs provide different experimental data and consequently different ANN weights and physichochemical parameters must be plugged to the system’s agent. ANNs, however, carry the flexibility to link different input types with desired outputs, and as such various physicochemical properties can be utilized, constituting this digital twin as a universal predictive platform suitable for BCS II drugs [29], whereas the iterated processes are examined. Pharma 4.0 capacitating digital twins fused with artificial wisdom will in the future decrease the need for the implementation of experiments and exploit real time manufacturing process data towards the constant adjustment of the tuning parameters, succeeding the control and prediction of the process’s progress and material’s quality attributes in time. Although fully functional digital twins require demanding computational and sensoring infrastructure as continuous curve fitting, probabilistic forecast, information evaluation and system configuration are taking place simultaneously, their multimodal capabilities shall be undeniably useful for industry deployment in the years to come.

## Figures and Tables

**Table 1:** PC-SAFT parameters of EO and PO groups for the calculation of the Poloxamers copolymers’ corresponding ones.

Group	*m_seg_* (−)	*σ_i_* (A)	*u_i_*/*k* (K)	Source
EO	0.052 *ΜW_total_*	2.89	206.74	[10,11,12,13,14]
PO	0.037 *MW_total_*	3.34	192.72	

**Table 2:** Discrete experimental sampling values used as training data for the ANN. These values represent three performed experiments using Itraconazole as API and Poloxamer-188 as stabilizer [19], which proved to be the most suitable after the Gibbs energy analysis.

Time (s)	Experiment 1 D50 (μm)	Experiment 2 D50 (μm)	Experiment 3 D50 (μm)
360	1.39	1.31	1.37
720	0.874	0.792	0.846
1440	0.784	0.742	0.783
2160	0.522	0.501	0.520
2880	0.467	0.434	0.436
3600	0.305	0.297	0.301

**Table 3:** Input and output parameters considered for the development of the digital twin.

MODEL	PARAMETER	TYPE	VALUE	UNIT
	Water quantity	input	9	mL
	API content	input	0.5	g
	Stabilizer content	input	0.25	g
	Mannitol content	input	1	g
Wet mill	Initial particle size (D50)	input	1.5	μm
	Grinding time	input	1	h
	Rotor speed	input	600	rpm
	Rotor diameter	input	40	mm
	Equipment volume	input	48	mL
	D50(t)	output	-	-
	Air temperature	input	110	°C
	Air flow	input	800	L h^−1^
Spray dryer	Air pressure	input	5	bar
	Drying chamber volume	input	5	L
	Drying time	input	1	h
	Final product size (D50)	output	10	μm

**Table 4:** Interfacial Gibbs energy and material properties correlation.

Stabilizer	$G_{m}^{i}$	Density (kg m^−3^)	Z-Potential
HPC-SL	0.0019	1320	−11.7
Poloxamer-407	0.0039	954	−13.7
Poloxame-188	0.0056	951	−17.0

## Data Availability

Not applicable.
